# Telitacicept treatment for recurrent IgA nephropathy after kidney transplantation

**DOI:** 10.1093/ckj/sfaf232

**Published:** 2025-07-16

**Authors:** Lichen Xu, Shukun Wu, Ping Zhang, Fang Wang, Wenjia Di, Shan Zhong, Yifu Hou, Hongji Yang, Guisen Li

**Affiliations:** Department of Nephrology and Institute of Nephrology, Sichuan Provincial People's Hospital, School of Medicine, University of Electronic Science and Technology of China, Chengdu, China; Department of Nephrology and Institute of Nephrology, Sichuan Provincial People's Hospital, School of Medicine, University of Electronic Science and Technology of China, Chengdu, China; Department of Nephrology and Institute of Nephrology, Sichuan Provincial People's Hospital, School of Medicine, University of Electronic Science and Technology of China, Chengdu, China; Department of Nephrology and Institute of Nephrology, Sichuan Provincial People's Hospital, School of Medicine, University of Electronic Science and Technology of China, Chengdu, China; Transplantation Center, Sichuan Provincial People's Hospital, School of Medicine, University of Electronic Science and Technology of China, Chengdu, China; Transplantation Center, Sichuan Provincial People's Hospital, School of Medicine, University of Electronic Science and Technology of China, Chengdu, China; Transplantation Center, Sichuan Provincial People's Hospital, School of Medicine, University of Electronic Science and Technology of China, Chengdu, China; Transplantation Center, Sichuan Provincial People's Hospital, School of Medicine, University of Electronic Science and Technology of China, Chengdu, China; Department of Nephrology and Institute of Nephrology, Sichuan Provincial People's Hospital, School of Medicine, University of Electronic Science and Technology of China, Chengdu, China

**Keywords:** IgA nephropathy, kidney transplantation, recurrence, telitacicept

## Abstract

**Background:**

Immunoglobulin A nephropathy (IgAN) is frequently recurrent after kidney transplantation, posing significant challenges in management. Current treatments, including glucocorticoids and immunosuppressants, have shown limited effectiveness in treating recurrent IgAN. A phase 2 clinical trial indicated that telitacicept could reduce proteinuria in patients with primary IgAN. In this report, we conduct a retrospective analysis to assess the efficacy and safety of telitacicept in treating recurrent IgAN among kidney transplant recipients.

**Methods:**

A retrospective cohort study was conducted from August 2023 to April 2025. Patients with biopsy-proven recurrent IgAN following kidney transplantation who were treated with telitacicept were included. Clinical data were collected from hospitalization records and outpatient follow-ups. The primary outcome was proteinuria reduction at 6 months, with extended evaluation at 12 months. Renal function changes were also observed.

**Results:**

Ten patients with recurrent IgAN were treated with telitacicept. After a 6-month follow-up, two patients achieved complete remission (CR), and two patients reached partial remission (PR). Furthermore, six patients (60%) experienced a reduction of over 30% in proteinuria by the end of the 6-month treatment period. At 9-month follow-up, one patient reached CR, two patients reached PR and five patients (50%) showed a reduction in proteinuria. By the 12-month follow-up, serum creatinine levels and estimated glomerular filtration rate remained stable in nine patients. Furthermore, the treatment also effectively reduced urine red blood cell counts.

**Conclusions:**

Telitacicept shows promising safety and efficacy in lowering proteinuria for patients with recurrent IgAN following kidney transplantation.

KEY LEARNING POINTS **What was known:**

- Immunoglobulin A nephropathy (IgAN) is frequently recurrent after kidney transplantation, posing significant challenges in management.

**This study adds:**

- In this report, we conduct a retrospective analysis to assess the efficacy and safety of telitacicept in treating recurrent IgAN among kidney transplant recipients.

**Potential impact:**

- In conclusion, telitacicept shows promising safety and efficacy in lowering proteinuria for patients with recurrent IgAN following kidney transplantation.

## INTRODUCTION

The long-term prognosis of immunoglobulin A nephropathy (IgAN) is not as optimistic as once believed [1–4]. Recently, a large IgAN cohort was followed-up and the outcomes of these patients with IgAN are poor with few of them expected to avoid kidney failure in their lifetime [2]. A large cohort of IgAN patients in China revealed that those with proteinuria levels >0.5 g/day have a significantly increased risk of kidney failure, which is defined as a composite of a 50% reduction in estimated glomerular filtration rate (eGFR) or progression to end-stage kidney disease [3].

Numerous studies suggest that patients with IgAN who progress to end-stage kidney disease have a high risk of experiencing recurrent IgAN after receiving kidney transplantation [5–8]. Unlike nontransplant primary IgAN patients, kidney transplant recipients are being treated with corticosteroid and immunosuppressants (including tacrolimus and mycophenolic acid analogs), therefore, many treatments for IgAN often fail to yield satisfactory results [9]. Patients with recurrent IgAN are more prone to severe proteinuria and rapid disease progression, which can ultimately lead to a loss of graft function.

Although new therapies are rapidly emerging that target different pathways, such as nefecon (a targeted release of budesonide) [10], sparsentan [a dual blocker of Endothelin type A (ETA) and Angiotensin type 1 receptor (AT1R)] [11], sibeprenlimab (a humanized IgG2 monoclonal antibody) [12] and iptacopan (a complement factor B inhibitor) [13], all of which have shown significant advancements in treating primary IgAN, their effectiveness in addressing recurrent IgAN following kidney transplantation remains unclear. In a single-center retrospective study, Gandolfini *et al*. [14] reported no evidence of urinary protein/creatinine ratio reduction or eGFR slope stabilization in 10 kidney transplant recipients with recurrent IgAN treated with targeted-release budesonide.

The widely recognized multi-hit model of IgAN pathogenesis involves key steps: production of circulating galactose-deficient IgA1, immune complexes deposition and kidney injury [15]. A significant aspect of this model is the activation of B cells, which includes their priming in the gut mucosa and the increased levels of cytokines such as B cell activating factor (BLys/BAFF) and a proliferation-inducing ligand (APRIL) [16]. The activated B cells play a vital role in the development of IgAN.

B-cell targeted therapy has shown promising results in the treatment of IgAN, by utilizing nefecon to inhibit B-cell priming in the gut mucosa [10], as well as employing BAFF and APRIL antibodies to suppress B cell activation [12, 17, 18]. It is essential to investigate whether B-cell targeted therapy can enhance the prognosis of recurrent IgAN following transplantation. Anti-CD20 antibodies may be crucial in managing post-transplantation recurrent IgAN. A previous study demonstrated that a rituximab-based regimen significantly reduced proteinuria within 12 months and sustained renal allograft function—the primary endpoint—for nearly 3 years [19].

Telitacicept is a fusion protein that effectively inhibits the activation of both BAFF and APRIL. A phase 2 clinical study shows that telitacicept can significantly reduce proteinuria in patients with IgAN [17]. It has confirmed that telitacicept has favorable efficacy and safety for IgAN patients with moderate proteinuria and renal insufficiency. A multicenter study of 97 IgAN patients showed telitacicept—alone or combined with steroids—significantly and safely reduced proteinuria after 3-month follow-up [20].

Can telitacicept decrease proteinuria and improve outcomes in patients with recurrent IgAN after renal transplantation? In this report, we present a retrospective analysis aimed at evaluating the efficacy and safety of telitacicept in treating recurrent IgAN in patients with kidney transplantation. This study could offer a promising new treatment option for patients suffering from recurrent IgAN.

## MATERIALS AND METHODS

### Patients

This study conducted a retrospective study on patients diagnosed with recurrent IgAN following kidney transplantation at Sichuan Provincial People's Hospital, spanning from 1 August 2023 to 30 April 2025. The recurrent IgAN after kidney transplantation was diagnosed by renal biopsy pathology and categorized according to the Oxford IgAN pathological criteria. The study included patients who were treated with corticosteroids and immunosuppressants post-transplantation, with an additional treatment of telitacicept. All enrolled patients optimally treated with non-immunosuppressive antiproteinuric agents before initiating telitacicept.

The inclusion criteria were as follows: (i) participants aged between 18 and 70 years; (ii) patients diagnosed with IgAN via biopsy of the transplanted kidney and accepted treatment of telitacicept; (iii) blood pressure maintained below 140/90 mmHg; (iv) patients who underwent regular follow-ups after kidney transplantation.

The exclusion criteria were as follows: (i) secondary IgAN; (ii) patients undergoing multiple organ or combined organ transplantation; (iii) those with complications of liver failure or heart failure; (iv) patients who irregularly adhered to their medication regimen; (v) individuals diagnosed with nephrotic syndrome, crescentic nephritis (with crescents exceeding 50%), or minimal change nephropathy associated with IgA deposition.

Clinical data were collected from both hospitalization records and outpatient follow-ups. This study complies with the principle of protecting the rights and interests of subjects in the Declaration of Helsinki and has been approved by the Ethics Committee of Sichuan Provincial People's Hospital [No.: (2025) Clinical Review No. (6)].

### Treatment

Conventional treatment involves the administration of tacrolimus and mycophenolate mofetil (or sodium mycophenolate), along with corticosteroids as the immune maintenance regimen. Additionally, some patients received a combination of angiotensin-converting enzyme inhibitors or angiotensin II receptor blockers, as well as sodium-glucose cotransporter-2 inhibitors for supportive care. For patients whose blood pressure fails to achieve the target of <140/90 mmHg, antihypertensive drugs were added.

The initial dosage of telitacicept for enrolled patients is 160 mg, administered subcutaneously once a week. Subsequent doses may be adjusted up to 240 mg weekly, depending on individual patient needs, including considerations for adverse reactions and disease progression.

### Data collection and efficacy evaluation

We collected data from patients before treatment, which included information on age, gender, weight, ethnicity, systolic and diastolic blood pressure, as well as information about renal transplantation. We also collected their medical history, noting any comorbidities and complications such as hypertension, diabetes, cardiovascular disease, liver failure, and tuberculosis. Additionally, various laboratory parameters were recorded, including complete blood count, CRP levels, 24-h urinary protein excretion, serum creatinine (Scr), eGFR, serum albumin, uric acid, liver function, blood lipid profiles and other biochemical markers. We monitored changes in 24-h urinary protein excretion, hematuria, serum albumin, Scr and eGFR at 3, 6, 9 and 12 months. Furthermore, we reviewed any related adverse events during this period.

The primary outcome was proteinuria reduction at 6 months, with extended evaluation at 12 months. Renal function (including Scr and eGFR) changes were also evaluated. If 24-h urinary protein excretion data is not available, the urine albumin/creatinine ratio will be used as a surrogate. Complete remission (CR) is defined as having 24-h urinary protein excretion of less than 0.3 g/day and an increase of Scr less than 15%. Partial remission (PR) occurs when there is a decrease in 24-h urinary protein excretion of 50% or more (not exceeding 3.5 g/day) compared with the baseline, along with an increase in Scr of <15%. Non-remission is classified as failing to meet any of the criteria mentioned above.

Secondary outcomes were defined as the change in Scr and eGFR during the follow-up period relative to the baseline. Safety evaluation included the incidence and severity of adverse events. Serious adverse events were defined according to International Council for Harmonisation Good Clinical Practice (ICH-GCP) guidelines as any untoward medical occurrence that: (i) resulted in death; (ii) was life-threatening; (iii) required inpatient hospitalization or prolonged existing hospitalization; (iv) resulted in persistent or significant disability/incapacity; or (v) required medical or surgical intervention to prevent any of these outcomes. All serious adverse events were monitored and documented throughout the study.

### Statistical methods

A normality test is performed on all data before statistical analysis. Normally distributed measurement data are expressed as mean ± standard deviation. Repeated measures analysis of variance is used for comparison before and after treatment. Non-normally distributed measurement data are expressed as median (p25, p75). Count data are expressed as number of cases and/or percentage. *P* < .05 is considered statistically significant.

## RESULTS

### Demographic and baseline clinicopathological characteristics of the patients

Ten patients with biopsy-proven recurrent IgAN following kidney transplantation were included in this study. The demographic and baseline characteristics of all participants are detailed in Table 1. All enrolled patients had been consistently treated with glucocorticoids and immunosuppressants post-transplantation, along with an additional treatment of telitacicept (Table 1) (Supplementary data, Table S1). During follow-up monitoring, three patients discontinued treatment at 3 months, and two patients at 6 months. The remaining patients completed the 12-month telitacicept treatment. Treatment discontinuation due to insufficient therapeutic response occurred in three patients, as proteinuria demonstrated no clinically relevant changes from baseline to 3 months. Financial constraints (inability to afford the medication) accounted for two of the treatment discontinuations.

**Table 1:** Demographic and baseline characteristics of participants.

Characteristics	Participants
Female, *n* (%)	4 (40)
Age, years	34.6 ± 11.8
Body mass index, kg/m^2^	25.21 ± 4.37
Systolic blood pressure, mmHg	133 ± 6
Diastolic blood pressure, mmHg	85 ± 4
Proteinuria, g/day	2.92 ± 1.93
eGFR, mL/min/1.73 m^2^	46.1 ± 20.4
IgA, g/L	2.75 ± 0.33
IgG, g/L	8.05 ± 1.31
IgM, g/L	1.29 ± 0.18
C3, g/L	1.00 ± 0.21
C4, g/L	0.19 (0.18–0.33)
Undergoing systemic corticosteroid therapy, *n* (%)	10 (100)
Undergoing immunosuppressant therapy, *n* (%)	10 (100)
Diabetes mellitus, *n* (%)	1 (10)
Time from KT to recurrent IgAN, years	5.0 ± 2.6
Oxford pathological score, %	
M0/M1	100/0
E0/E1	100/0
S0/S1	25/75
T0/T1/T2	75/25/0
C0/C1	50/50

KT, kidney transplantation; M, mesangial hypercellularity score; E, endocapillary hypercellularity score; S, segmental glomerulosclerosis score; T, tubular atrophy fibrosis score; C, crescent score.

The mean age of the participants was 34.6 ± 11.8 years, and 40% were women. The median duration from kidney transplantation to diagnosis of recurrent IgAN is 5.0 ± 2.6 years. The mean body mass index was 25.21 ± 4.37 kg/m^2^. Blood pressure was well controlled throughout the study, averaging 133 ± 6/85 ± 6 mmHg.

With respect to renal allograft histopathology, according to the MEST-C criteria in the updated Oxford classification of IgAN, none of the patients had a mesangial cell score (M0) and no diffuse endocapillary hypercellularity (E0). Six patients had segmental glomerulosclerosis or adhesion (S1), two patients had tubular atrophy or interstitial fibrosis (T1) and four patients had cellular or fibro-cellular crescents (C1). The detailed data from the renal biopsy was unavailable for two patients.

### Changes of proteinuria and renal function after treatment with telitacicept

**Figure 1:**
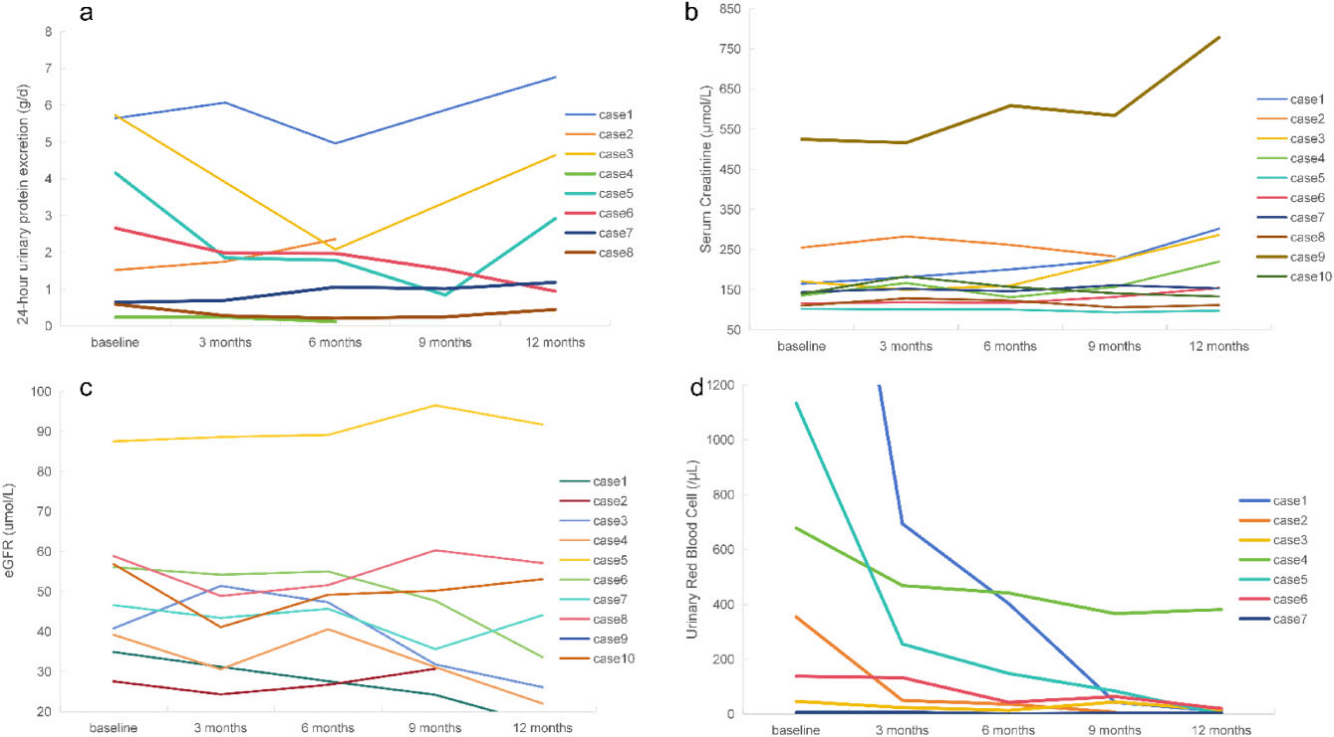
Changes in proteinuria, Scr, eGFR and urinary RBC counts from baseline to 12 months in patients receiving telitacicept; (**a**) 24-h proteinuria, (**b**) Scr, (**c**) eGFR, (**d**) urinary RBC counts.

In Fig. 1, we present the trajectories of the parameters following telitacicept treatment. After 6 months, two patients (20%) achieved complete remission, while another two patients (20%) achieved partial remission. Notably, eight patients (80%) demonstrated a declining trend in proteinuria (Fig. 1a). Furthermore, six patients (60%) experienced a reduction of over 30% in proteinuria by the end of the 6-month treatment period. At 9-month follow-up, one patient reached CR, two patients reached PR and five patients (50%) exhibited a declining trend in proteinuria.

The mean 24-h proteinuria level declined significantly from a baseline of 2.92 ± 1.93 g/day to 1.66 ± 1.59 g/day (−43.15%) after 3 months of treatment. A partial rebound occurred by 6 months (1.96 ± 1.87 g/day, −32.88% vs baseline), followed by a further reduction at 9 months (1.72 ± 1.87 g/day). However, after 12 months of treatment, proteinuria increased to 3.11 ± 2.33 g/day (Table 2). Notably, the average serum albumin levels showed a gradual increase over time, with a 8.86% elevation from baseline observed at the 12-month treatment.

**Table 2:** Parameters and changes of laboratory parameters in enrolled patients.

Parameters	Baseline	3 months	6 months	9 months	12 months
WBC, 10^9^/L	8.94 ± 3.40	10.94 ± 7.04	9.66 ± 3.32	9.90 ± 3.70	9.85 ± 2.73
Lymphocyte, 10^9^/L	1.79 ± 1.04	2.73 ± 1.06	2.72 ± 0.94	2.34 ± 1.06	2.6 ± 1.05
Neutrophil, 10^9^/L	6.58 ± 3.58	7.42 ± 6.48	6.21 ± 2.52	7.00 ± 3.31	6.39 ± 1.96
RBC, 10^12^/L	4.66 ± 0.83	4.63 ± 0.93	4.69 ± 0.95	4.82 ± 1.19	4.75 ± 1.07
Hemoglobin, g/L	132.6 ± 23.2	129.9 ± 23.1	132.1 ± 22.8	133.9 ± 30.9	133.6 ± 21.6
Albumin, g/L	39.75 (37.13–45.53)	44.75 (43.07–46.63)	44.60 (41.98–45.90)	45.05 (41.88–47.93)	43.90 (40.15–45.45)
ALT, U/L	17.1 ± 6.4	19.8 ± 11.4	22.8 ± 21.3	19.6 ± 8.1	20.0 ± 7.8
TBIL, μmol/L	10.3 ± 5.5	7.6 ± 4.6	8.3 ± 4.5	9.0 ± 3.4	7.1 ± 2.1
Platelet, 10^9^/L	187 (167–217)	184 (109–245)	164 (129–220)	180 (163–300)	204.78 ± 94.28
BUN, mmol/L	8.4 (5.9–12.7)	9.5 (7.5–11.9)	7.7 (6.5–11.9)	8.3 (6.6–11.9)	9.9 (6.3–13.1)
Uric acid	338.3 ± 117.9	371.9 ± 66.6	362.7 ± 97.7	330.5 ± 66.4	336.6 ± 78.7
eGFR, mL/min/1.73 m²	46.1 ± 20.4	42.7 ± 20.7	44.3 ± 21.0	41.8 ± 23.9	39.2 ± 25.6
SCr, μmol/L	141.9 (114.6–191.6)	159.6 (126.1–207.9)	151.5 (121.5–215.8)	159.1 (125.5–226.0)	154.3 (122.1–294.3)
Proteinuria, g/day	2.92 ± 1.93	1.66 ± 1.59	1.96 ± 1.87	1.72 ± 1.87	3.11 ± 2.33
URBC, /μL	91.9 (5.2–792.0)	36.6 (4.8–308.4)	24.6 (3.5–211.4)	39.9 (4.5–68.6)	12 (7–200.4)

WBC, white blood cell; ALT, alanine aminotransferase; TBIL, total bilirubin; BUN, blood urea nitrogen; URBC, urinary red blood cell count.

As for secondary outcomes, the baseline Scr was 141.9 (114.6–191.6) μmol/L, and the eGFR was 46.1 ± 20.4 mL/min/1.73 m². The Scr and eGFR values of the nine patients almost remained stable during treatment (Fig. 1b and c). The eGFR decreased to 44.3 ± 21.0 mL/min (with a decrease of 3.84%) and Scr increased to 151.5 (121.5–215.8) μmol/L (with an increase of 6.77%) after 6 months of treatment (*P* > .05). Treatment monitoring revealed progressive renal function changes, with measurable declines in eGFR and concurrent elevations in Scr at both the 9- and 12-month timepoints. Follow-up evaluations revealed moderate eGFR reductions at 9 and 12 months post-treatment, with stable mean Scr levels maintained during this period.

### Changes in other laboratory parameters

In particular, after 12 months of treatment, there was a significant remission of hematuria (Fig. 1d). The urine red blood cell count decreased from 91.9 (5.2–1792.0) PCS/μL to 36.6 (4.8–1308.4) PCS/μL after 3 months, and to 24.6 (3.5–1211.4) PCS/μL after 6 months (with a decrease of 73.23%) of treatment. There are four patients ≤5 red blood cells (RBC)/high-power field using the manual method or ≤28 RBC/μL using the automated method after treatment of 6 months. By the 12-month treatment endpoint, the mean hematuria level demonstrated sustained reduction, achieving an 86.94% decrease from baseline values.

Regarding serum uric acid, after 6 months of treatment, the average serum uric acid levels of 10 patients showed an upward trend, serum uric acid of 1 patient increased more than 30%, 3 patients had an increase of 10%–30% and 4 patients showed a downward trend. Nevertheless, comprehensive assessment after 12 months of treatment, the mean serum uric acid levels remained stable (338.3 ± 117.9 to 336.6 ± 78.7).

### Safety of telitacicept in renal transplant recipients

After 6 months of treatment, telitacicept increased the serum levels of leukocytes compared with baseline (8.05%). The mean change in lymphocytes was increasing 51.96% (*P* < .05), and the mean change in neutrophils was decreasing 5.96%. At the 12-month treatment, the serum levels of leukocytes showed minimal change compared with 6-month levels but remained elevated by 10.18% from baseline. The mean change in lymphocyte increased significantly by 45.25% from baseline, while neutrophils remained stable (6.58 ± 3.58 vs 6.39 ± 1.96, baseline vs 12 months). Among other serologic biomarkers, the mean serum level of alanine aminotransferase and total bilirubin remained stable after treatment of telitacicept after 12 months.

During treatment, no participants died, no events of kidney failure requiring kidney replacement therapy occurred, and no participants progressed to end-stage kidney disease. One patient had diarrhea, one patient had upper respiratory tract infection and one patient had urinary tract infection during treatment. Two patients had symptoms of dizziness and nausea after injection, which could be relieved spontaneously. Furthermore, one patient developed antibody-mediated rejection after 12 months of treatment (Table 3).

**Table 3:** Adverse events during treatment period.

Events, *n* (%)	Participants
AE, *n* (%)	4 (40)
Serious AE, *n* (%)	0 (0)
AE resulting in reduction or temporary discontinuation of telitacicept, *n* (%)	1 (10)
AE resulting in discontinuation of telitacicept, *n* (%)	0 (0)
Death, *n* (%)	0 (0)
Antibody-mediated rejection, *n* (%)	1 (10)
Hyperuricemia, *n* (%)	4 (40)
Upper respiratory tract infection, *n* (%)	1 (1)
Urinary tract infection	1 (1)
Injection-site reactions	2 (20)
Dizziness, *n* (%)	2 (20)
Diarrhea, *n* (%)	1 (10)

AE, adverse events.

## DISCUSSION

The recurrent IgAN after kidney transplantation poses a significant threat to the transplanted kidney, manifesting hematuria, proteinuria, with or without increased Scr [21–23]. Renal biopsy pathology serves as the primary diagnostic method. Given that these kidney transplant patients are undergoing a combination therapy of corticosteroids and immunosuppressants (medications also employed to manage IgAN at risk of progression), it indicates that these recurrent IgAN are resistant to such treatments. This report showed that telitacicept has demonstrated promising efficacy and safety in treating recurrent IgAN in kidney transplant recipients.

In recent years, the success rates and outcomes of kidney transplantation have significantly improved, primarily due to advancements in new immunosuppressive drugs that selectively inhibit key molecular signals, as well as ongoing research into the biological processes underlying graft injury. Telitacicept is a protein created by fusing the specific soluble extracellular domain of TACI with the human IgG1 Fc segment using recombinant DNA technology [24]. This innovative protein exhibits a dual-target effect: it inhibits the maturation and differentiation of B cells while also reducing the generation of plasma cells. Consequently, it decreases the formation of plasma cells that produce IgA1 and reduces the production of galactose-deficient IgA1 (Gd-IgA1) dimers [25–27]. Additionally, telitacicept inhibits the production of IgG antibodies against Gd-IgA1 [28], leading to a reduction in immune complex deposition and improved IgA deposition within the glomerular mesangial area [29–31]. This multifaceted approach effectively achieves a comprehensive inhibition of B-cell maturation and differentiation.

In a phase 2b trial for patient with systemic lupus erythematosus (SLE) at Week 48, the telitacicept group had conspicuously better effectiveness in achieving an SLE Responder Index 4, rather than the placebo group (*P* < .001) [32, 33]. Our study shows that after 6 months of treatment with telitacicept, it has significant effectiveness—over half of patients showed >30% decrease in proteinuria, with a stable level of eGFR and Scr. Despite treatment discontinuation in some patients (which may have contributed to the overall increase in mean proteinuria), one patient achieved CR and two attained PR after 9-month treatment. In addition, this study also found that the level of urine RBC in patients was significantly relieved. It may reflect the improvement of inflammation and bleeding in the glomeruli.

Regarding safety, telitacicept was generally well tolerated in patients with SLE in several previous studies [34, 35]. In previous clinical trials, almost all the observed side effects of telitacicept were mild to moderate [17]. In this study, no severe adverse events were found during treatment of 12 months. It could be observed the increase of lymphocytes, Zhao have found that treatment with telitacicept was associated with an increase in the absolute values of CD19+, CD27+ and IgD+ B cells, and then subsequently decreased to baseline or lower than baseline levels [36]. This indicates that a longer follow-up period may be required for further assessment of its safety.

This study has several limitations. First, as a retrospective analysis, it is inherently susceptible to biases such as patient selection bias, missing data, and unmeasured confounding factors, thereby limiting causal inference. Second, because all enrolled patients received glucocorticoids and immunosuppressants, no control group was included, which may reduce the generalizability of our findings. Third, incomplete 24-h proteinuria excretion data for some participants could affect the reliability of related outcomes. Finally, we did not assess post-treatment changes in key biomarkers—including IgA, IgM, IgG, BAFF/APRIL and Gd-IgA1—following telitacicept therapy.

Future research is necessary to investigate the differences in the efficacy of telitacicept at varying dosages and frequencies. Additionally, the impact of telitacicept on circulating Gd-IgA1 or IgA1 complexes needs further evaluation. In addition, economic barriers highlight challenges in real-world drug accessibility, future research should integrate socioeconomic support strategies to enhance retention.

## CONCLUSIONS

Telitacicept shows promising safety and effectiveness in the recurrence of IgAN in patients after kidney transplantation. It effectively reduces levels of proteinuria and urinary RBC. Furthermore, throughout the treatment, Scr levels and eGFR remain stable in the majority of patients. It provides new treatment for enhancing the long-term prognosis of patients with recurrent IgAN and presents potential opportunities for addressing other autoimmune diseases where B lymphocytes are significantly involved in the disease process.

## Supplementary Material

sfaf232_Supplemental_File

## Data Availability

The data analyzed or generated during the study are available from the corresponding author on reasonable request.
