# Supplementary material for: Telitacicept treatment for recurrent IgA nephropathy after kidney transplantation
Source: Clin Kidney J. 2025 Jul 16;18(8):sfaf232. doi: 10.1093/ckj/sfaf232 (PMC12343103; doi:10.1093/ckj/sfaf232)
Supplement: sfaf232_Supplemental_File [file sfaf232_supplemental_file.docx]

Supplementary Table 1 The basic information and treatment of the enrolled patients

| **No.** | **Age** | **Gen-**  **der** | **MEST-C Score** | **Time of onset** | **Time of kidney transplantation** | **Time of Renal biopsy** | **Time of enrollment** | **Glucocorticoids** | **Immunosuppressant** | | | |
| --- | --- | --- | --- | --- | --- | --- | --- | --- | --- | --- | --- | --- |
|  |  |  |  |  |  |  |  |  | **Tacrolimus** | **Mycophenolate Sodium** | | **MMF** |
| 1 | 37 | F | M0E0S1T0C1 | 2018 | 2020.02 | 2023.08 | 2023/08 | 10mg qd | 1mg qd  0.5mg qn | | 360mg qd  180mg qn |  |
| 2 | 33 | M | M0E0S1T1C1 | 2017 | 2018.06 | 2023.12 | 2023/12 | 5mg qd | 2mg bid | | 360mg bid |  |
| 3 | 35 | M | Unavailable | 2013 | 2019 | 2024.01 | 2024/02 | 10mg qd | 1mg qd  0.5mg qn | | 540mg qd  360mg qn |  |
| 4 | 50 | F | MOE0S1T1C0 | 2011 | 2014 | 2024.03 | 2024/03 | 5mg qd | 0.25mg bid | |  | 1g qd  0.5g qn |
| 5 | 25 | M | Unavailable | 2017 | 2017.10 | Unavailable | 2024/03 | 10mg qd | 2mg qd  1.5mg qn | | 540mg qd  360mg qn |  |
| 6 | 27 | F | M0E0S0T0C1 | 2018 | 2023.09 | 2024.04 | 2024/04 | 7.5mg qd | 1.5mg bid | | 540mg bid |  |
| 7 | 56 | M | MOE0S1T0C1 | 2011 | 2019.02 | 2024.04 | 2024/04 | 5mg qd | 1.5mg bid | | 360mg bid |  |
| 8 | 28 | F | M0E0S0T0C0 | 2017 | 2018.09 | 2024.04 | 2024/04 | 10mg qd | 1.5mg bid | | 540mg bid |  |
| 9 | 20 | M | M0E0S1T0C0 | 2021 | 2022 | 2023.09 | 2024/05 | 5mg qd | 3mg bid | | 540mg bid |  |
| 10 | 33 | M | MOE0S1T0C0 | 2018 | 2018.08 | 2024.05 | 2024/05 | 5mg qd | 0.5mg qd | | 540mg bid |  |

F, female; M, male; MMF, Mycophenolate Mofetil
